# Adapting a first- and second-year medical student psychiatric interview course for virtual learning

**DOI:** 10.12688/mep.18946.1

**Published:** 2022-05-10

**Authors:** Ashley Curry, Austin Butterfield, Leigh Kunkle, Joseph Sakai

**Affiliations:** 1 Department of Psychiatry, University of Colorado, Anschutz Medical Campus, Aurora, CO, 80045, USA; 2 Denver Health, Denver, CO, 80204, USA

**Keywords:** medical education, telehealth, psychiatric interview, virtual learning, curriculum development

## Abstract

**Background**: In response to the COVID-19 pandemic, a first- and second-year psychiatric interviewing course was converted to a virtual platform with interviews performed via video conferencing. Telepsychiatry has been shown to be an effective modality for patient care, but little is known about the effectiveness of using this modality to teach the psychiatric interview. We sought to examine how switching to remote learning would affect the quality of the course.

**Methods**: We compared student course evaluations from 2019 (in-person) with evaluations from the 2020 (virtual). Using Likert scales, students were asked to rate their comfort in interviewing patients, discussing emotional and psychological topics, and documenting the encounter. Student responses were supplemented with qualitative feedback and input from faculty facilitators.

**Results**: We found no significant difference in student reports of their overall experience with the course, comfort with interviewing patients or with discussing emotional and psychological issues. The course reduced student self-reported stigma toward mental illness.

**Conclusions**: The virtual delivery of this course offers learners a very similar experience to an in-person course. Looking beyond the pandemic, this model could have applications in other institutions where geographic or other logistical considerations would impede the implementation of such a course in-person.

## Introduction

The COVID-19 pandemic has led to the rapid virtualization of healthcare, and with it, the need for transformation in the delivery of medical education ^1, 2^. In response to the COVID-19 pandemic and in accordance with guidelines from the American Association of Medical Colleges, in March 2020 all non-clinical learning was transitioned online and clinical rotations were paused. While clinical rotations have since resumed, many students are participating in telehealth visits for the first time, and most teaching in the pre-clinical years remains remote. Telehealth training was an emerging theme in medical school curricula prior to the COVID-19 pandemic ^3^, but current circumstances underscore the need to educate future physicians to provide care through virtual modalities.

At the University of Colorado, we endeavored to create a virtual offering of our Basic Psychiatry Sequence for first- and second-year medical students. This 15-session course traditionally combined didactic learning and live interviews of volunteers with mental illness. Students were introduced to psychiatric diagnoses and gained practical experience communicating with patients. Both standardized and real patient interactions have been found to be effective in teaching the psychiatric interview skills to medical students ^4, 5^, but there is little evidence regarding the effectiveness of using these modalities in a virtual setting.

While telepsychiatry was never previously a focus of this course, the established efficacy of telepsychiatry in a variety of clinical settings ^6^ suggested that virtual interviews with patients would still allow for robust learning with regard to psychiatric diagnosis and communication skills. Telepsychiatry experiences are well-received by medical students ^7^, but little research exists on training medical students in telepsychiatry ^8^. In this paper, we describe our experience with the development and assessment of a virtual psychiatry course for first- and second-year medical students. The effect of this course on students’ perceptions of mental illness has been described previously but the difference between in-person and virtual offerings of the course has never been studied ^9^. 

### Course overview

The Basic Psychiatry Sequence course is a 15-session course imbedded in the curriculum for other organ systems. The course spans two academic years, commencing in the spring of the first year of medical school and concluding in the fall of second year, but each iteration of the course takes place in a single calendar year. Students are taught about various psychiatric conditions and diagnostic skills in a lecture and then meet in small groups (n=8–9) to interview a community volunteer who has lived experience with mental illness. Each small group is facilitated by two faculty members in psychiatry and behavioral health (employee or volunteer with MD, PhD or LCSW level of training). After the interviews, the group members discuss the case and peers and faculty give feedback on the interview. Students then practice documenting the encounter and submit written reflections of their experience. Each iteration of the course involves approximately 179–190 students, 43 faculty members (lecturers and small group facilitators) and over 150 community volunteers. The content of the course is described in Table 1.

**Table 1.** Description of weekly curriculum in the basic psychiatry sequence.

Week	Lecture Topic	Small-Group Interview Topic
1	Introduction to Psychiatry	Introduction to small groups (no interview)
2	Depression	Depressive disorders
3	Anxiety	Anxiety disorders
4	Suicide	Cardiac, pulmonary and/or renal problems and depression/anxiety
5	Post-traumatic stress disorders	Trauma-related disorders
6	Developmental disorders	Autism (volunteers or their caregivers)
7	Delirium, dementia and brain injury	Cognitive impairment (volunteers or their caregivers)
8	Somatic complaints and pain	Chronic pain
9	Anxiety disorders	Anxiety disorders
10	Bipolar and psychotic disorders	Bipolar or psychotic disorders
11	Psychotic disorders	Psychotic disorders
12	Substance use disorders	Substance use disorders
13	Illness in physicians	Physicians in the Colorado Physician Health Program
14	Eating disorders	Eating disorders
15	Thyroid or endocrine-related depression	Group wrap-up (no interview)

## Methods

The 2020 course transitioned to virtual learning approximately two weeks prior to its scheduled start. The course was quickly adapted to allow lectures and interview sessions to take place via secure video conferencing. The general structure of the course was preserved with students, faculty and volunteers joining from their homes (or other designated workspace) for lectures and small groups sessions.

Students and faculty facilitators completed evaluations at the conclusion of each year of the course. For student evaluations, every student who completed the course was required to complete a course evaluation. Facilitator evaluations were not mandatory. We compared results from the 2020 course (virtual) with the 2019 (in-person). Primary outcomes were comfort discussing psychological topics with patients, reduction in bias or stigma toward patients with mental health concerns, comfort interviewing psychiatric patients, comfort with documenting an encounter with a patient with mental illness and confidence in interviewing skills. Additional questions in the evaluations assessed the quality of the learning experience (quality of the small groups, ability to express ideas and contribute to the group, usefulness of feedback). Outcomes were assessed using either a five- or six-point Likert scale. Results were analyzed using chi-square analyses with statistical significance set at p < 0.05. For analysis, chi-square tests were performed on 2×2 tables with responses collapsed to the two highest Likert-scale scores (i.e., strongly agree/agree) compared to the aggregate of all other responses. A summary of questions (including the Likert scales used), student responses and results is outlined in Table 2.

**Table 2.** Summary of student evaluations from the 2019 in-person course (n=188) and the 2020 virtual course (n=179).

	Disagree + strongly disagree ^a^	Somewhat disagree	Somewhat agree	Agree	Strongly agree	Agree + strongly agree	Sum of other responses	χ ^2 b^ p-value ^c^
**My comfort level in discussing emotional/psychological issues with patients increased.**								
2019	3	3	23	90	69	**159 (84.6%)**	**29 (15.4%)**	0.121 p=0.73
2020	3	1	26	77	72	**149 (83.2%)**	**30 (16.8%)**	
**The small group sessions reduced biases or stigma I had toward patients with mental health concerns.**								
2019	10	6	42	68	62	**130 (69.1%)**	**58 (30.9%)**	0.067 p=0.80
2020	6	12	35	69	57	**125 (70.4%)**	**53 (29.6%)**	
	Not at all comfortable	Somewhat comfortable	Mostly comfortable	Very comfortable	Completely comfortable	Very comfortable + completely comfortable	Sum of other responses	χ ^2^ p-value
**How comfortable do you feel interviewing patients about psychiatric conditions covered in this course?**								
2019	1	23	65	82	17	**99 (52.7%)**	**89 (47.3%)**	2.014 p=0.16
2020	1	14	83	62	19	**81 (45.3%)**	**98 (54.7%)**	
**How comfortable are you with generating appropriate documentation for patients with mental illness?**								
2019	7	37	78	53	13	**66 (35.1%)**	**122 ** **(64.9%)**	1.788 p=0.18
2020	5	26	73	64	11	**75 (41.9%)**	**104 ** **(58.1%)**	
	Not at all confident	Somewhat confident	Mostly confident	Very confident	Completely confident	Very + completely confident	Sum of other responses	χ ^2^ p-value
**How confident are you with interviewing patients with mental illness?**								
2019	2	36	73	62	15	**77 (41.0%)**	**111 (59%)**	0.085 p=0.77
2020	1	16	86	66	10	**76 (42.5%)**	**103 ** **(57.5%)**	
	Not at all	A little	Somewhat	Mostly	Always	Mostly + always	Sum of other responses	χ ^2^ p-value
**To what extent have you been able to express your ideas in a collegial way?**								
2019	0	4	10	77	97	**174 (92.6%)**	**14 (7.4%)**	0.272 p=0.61
2020	1	3	12	70	93	**163( 91.1%)**	**16 (8.9%)**	
**To what extent have you personally been able to promote the work of the group?**								
2019	2	13	36	83	54	**137 (72.9%)**	**51 (27.1%)**	1.12 p=0.29
2020	2	6	32	87	52	**139 (77.7%)**	**40 (22.3%)**	
**To what extent did you receive useful feedback?**								
2019	2	5	20	67	94	**161 (85.6%)**	**27 (14.4%)**	0.070 p=0.79
2020	1	7	16	42	113	**155 (86.6%)**	**24 (13.4%)**	
	Poor	Fair	Good	Very good	Excellent	Very good + excellent	Sum of other responses	χ ^2^ p-value
**What was the overall quality of your small group experience?**								
2019	1	7	21	57	102	**159 (84.6%)**	**29 (15.4%)**	0.008 p=0.93
2020	2	6	19	63	89	**152 (84.9%)**	**27 (15.1%)**	

^a^For questions with a 6-pt Likert scale, bottom two responses were collapsed for ease of reporting

^b^Chi-square computed with 2x2 comparing sum of top two Likert scores with sum of remaining categories

^c^Significance set at p=0.05

Faculty facilitators were asked to rate their experiences as a small group co-leader (excellent, very good, good, fair, poor) and the extent to which they agreed with the following statement (strongly agree, agree, disagree, strongly disagree): “I felt effective as a small group facilitator.” Facilitator surveys were not linked from 2019 to 2020, so we did not have the ability to pair results. Faculty members who were present during both years were asked to compare the experience of in-person small groups to the virtual small groups (much better, a little bit better, about the same, a little bit worse, much worse). This evaluation activity was reviewed by the Colorado Multiple Institutional Review Board (ID #APP001–1; 3/16/21) and determined not to be human-subject research; participant consent was not required.

## Results

Initially, 190 students were enrolled in the 2019 (in-person) course and 180 in the 2020 (virtual) course. A total of 188 students in 2019 and 179 students in 2020 completed the required coursework and course evaluation. See Table 2 for full description of results. We found no significant difference in any of the outcomes when comparing the two years. Most students felt that the course increased their comfort level with discussing emotional and psychological issues with patients (84.6% in 2019, 83.2% in 2020) and reduced bias or stigma toward patients with mental health concerns (69.1% in 2019, 70.4% in 2020). Fewer students reported feeling very or completely comfortable interviewing patients about psychiatric conditions (52.7% in 2019, 45.3% in 2020) and documenting the encounter (35.1% in 2019, 41.9% in 2020), but results were unchanged between the two years. Fewer than half the students reported feeling very or completely confident interviewing patients with mental illness by the end of the course (41% in 2019, 42.5% in 2020). Overall response to the course was very positive with most students reporting a very good or excellent experience with the course (84.6% in 2019, 84.9% in 2020). Students also felt they could express their ideas in a collegial way (92.6% in 2019, 91.1% in 2020), personally promote the work of the group (72.9% in 2019, 77.7% in 2020) and that they received useful feedback during the course (85.6% in 2019, 86.6% in 2020). More than 95% of students felt mostly (21/175), very (77/175) or completely (71/175) comfortable using the video conferencing software (four students did not respond).

Approximately 60% of faculty facilitators (20/33) completed the survey for the 2019 course compared with 76% (25/33) for the 2020 course. All participants reported feeling effective as small group facilitators in both years (100% strongly agree/agree). The number of group facilitators rating their experience as a small group co-leader as excellent or very good decreased in 2020 when compared with 2019 (84%, 21/25 vs. 95%, 19/20), but overall remained positive with no participants rating the experience as fair or poor. All participants felt mostly (4/25), very (14/25) or completely (7/25) comfortable with the video conferencing software. Among the faculty members who participated in both years of the course (76%, 19/25), nine faculty members rated the course as a little bit worse, six estimated it was about the same, and two felt it was a little bit better.

## Conclusion

Student comfort with interviewing patients and documenting the encounter remained low after completion of this course. However, this is expected in an introductory course. For many students, this course was their first opportunity to perform a psychiatric interview. Proficiency in areas of interviewing and documentation is a longitudinal learning objective that students are expected to work toward during their four years of medical school. Even then, the challenges and nuances of the psychiatric interview are only fully explored if a student elects to pursue psychiatry residency training. The course serves additional important functions: to provide exposure to patients with psychiatric conditions, increase student comfort in discussing emotional or psychological topics and reduce stigma. Our results suggest that both the in-person and virtual versions of this course accomplish those goals, while also providing a positive and collegial learning environment.

An important limitation of this study was our inability to trial both formats of the course on the same population. While the similarity of responses between the two years leads us to conclude that the student experiences were very similar, it is important to note that the students did not have a basis for comparison when evaluating the course. We also were not able to pair faculty responses between 2019 and 2020 to evaluate individual reactions to the course changes.

Prior to the COVID-19 pandemic, patient interest in telehealth services already exceeded availability, and recent care shifts due to the pandemic underscore the importance that physicians at all levels of training become comfortable providing care in a virtual setting ^1^. While some issues with technology were noted by both students and faculty members, the experience of the virtual course was positive for both groups. Lack of comfort with technology is one of the largest barriers to acceptance of telehealth ^10^, so early exposure to virtual platforms through a course such as this one could serve to reduce this barrier for future generations.

Our study suggests that a virtual offering of a psychiatric interviewing course is similar in quality to an in-person course. As social distancing requirements are being relaxed around the country, we have elected to keep the course virtual during this entire academic year in case of sudden changes in local or state restrictions in the event of another virus surge. While the course was adapted out of necessity for social distancing, the effectiveness of the virtual course has implications for other schools that may have limited space or access to local volunteers or faculty. In 2020, 53 medical schools reported having regional campuses, with an average of two regional campuses per school for the pre-clinical years ^11^. The number of branch campuses is expanding every year, with 14 new branches added in 2020 ^12^. A virtual course would allow for coordination across campus sites with the ability to pool faculty and volunteers regardless of geographic location. Just as telepsychiatry has been used to address workforce shortages in rural or resource-poor areas ^1^, a telepsychiatry interviewing course could offer a robust introduction to psychiatry for students in settings where access to mental health care is lower and stigma toward psychiatric conditions may be higher.

## Data availability

### Underlying data

Repository: Adapting a First- and Second-year Medical Student Psychiatric Interview Course for Virtual Learning: https://doi.org/10.17605/OSF.IO/ZXQG8 ^13^

This project contains the following underlying data:

 2020–2021_ClinPsychGroupEvals_DeID.xlsx (2020 student responses)

- ClinPsychGroupEvals_DeID.xlsx (2019 student responses)

- Faculty survey 3.16.2022.xlsx (2019 and 2020 faculty responses)

Data are available under the terms of the Creative Commons Attribution 4.0 International license (CC-BY 4.0).
