# Supplementary material for: Adapting a first- and second-year medical student psychiatric interview course for virtual learning
Source: MedEdPublish (2016). 2022 May 10;12:35. [Version 1] doi: 10.12688/mep.18946.1 (PMC9370079; doi:10.12688/mep.18946.1)
Supplement: Peer review history

# Peer review: Reviewer response for version 1

**DOI:** 10.21956/mep.20303.r32071

**Published:** 2022-06-24

**Authors:** Mariette Volschenk, Kanita Brits

**Affiliations:** 1 Centre for Health Professions Education, Faculty of Medicine and Health Sciences, Stellenbosch University, Cape Town, South Africa

The authors describe the use of telehealth modalities to enhance a virtual psychiatry course for first- and second-year medical students in response to the COVID-19 pandemic. It compares students’ in-person and virtual learning experiences, using course evaluation forms, with a view to determine whether virtual learning affects the quality of the course.

 The title is concise and conveys the focus of the article well. The abstract provides a clear picture of the rationale for the study, the methods used, and the key findings. The introduction is grounded in the literature and provides adequate context. The research methodology seems appropriate, the results are well-presented, and the study is sufficiently motivated. The conclusions are adequately supported by the results and indicate relevance in the field of health professions education.

 This work is important because it highlights the need to integrate newer competencies such as telehealth and telepsychiatry into undergraduate medical education to better prepare graduates for future healthcare delivery. The finding that students had a very similar experience between in-person and virtual modalities may encourage other institutions to explore similar strategies. Having said that, the reviewers agree with the authors that the inability to trial both formats of the course on the same cohort poses a significant limitation – specifically since some faculty members who were present during both years and were asked to compare the facilitation of in-person small groups to virtual small groups, indicated that they experienced facilitating learning on the virtual platform “a little worse” than in-person.

 Incorporation of qualitative approaches into future research on this topic may provide useful insights into individual student learning experiences and may also prove helpful in understanding why some faculty members had different experiences from students.

Have any limitations of the research been acknowledged?

Yes

Is the study design appropriate and does the work have academic merit?

Yes

Is the work clearly and accurately presented and does it cite the current literature?

Yes

If applicable, is the statistical analysis and its interpretation appropriate?

I cannot comment. A qualified statistician is required.

Are all the source data underlying the results available to ensure full reproducibility?

Yes

Are the conclusions drawn adequately supported by the results?

Yes

Are sufficient details of methods and analysis provided to allow replication by others?

Yes

Reviewer Expertise:

Technology enhanced learning, professional identity, curriculum design

We confirm that we have read this submission and believe that we have an appropriate level of expertise to confirm that it is of an acceptable scientific standard.

# Peer review: Reviewer response for version 1

**DOI:** 10.21956/mep.20303.r32037

**Published:** 2022-05-26

**Authors:** Martin M. Klapheke

**Affiliations:** 1 Psychiatry, University of Central Florida College of Medicine, Orlando, FL, USA

This well-designed and conducted study is clearly presented and could be readily replicated by others. The authors’ finding of no significant difference in student reports of overall experience (for in-person and virtual platforms) makes a fine contribution to the literature, especially as COVID-19 has spurred the use of virtual platforms in medical education.

 It would be optimal to learn more about why some faculty felt the course was “a little bit worse” using the virtual platform.

 Though not a focus of this study, it would also appear very possible to expand this course to give students individual formative feedback (e.g., for their educational portfolio) on performance in patient interviewing, consistent with the Association of American Medical Colleges' proposed Educational Professional Activity #1, Gather a history and perform a physical [and/or mental status] examination ^1^.

Have any limitations of the research been acknowledged?

Yes

Is the study design appropriate and does the work have academic merit?

Yes

Is the work clearly and accurately presented and does it cite the current literature?

Yes

If applicable, is the statistical analysis and its interpretation appropriate?

Yes

Are all the source data underlying the results available to ensure full reproducibility?

Yes

Are the conclusions drawn adequately supported by the results?

Yes

Are sufficient details of methods and analysis provided to allow replication by others?

Yes

Reviewer Expertise:

Research in medical education, including use of simulation.

I confirm that I have read this submission and believe that I have an appropriate level of expertise to confirm that it is of an acceptable scientific standard.

## References

[1] : Core Entrustable Professional Activities for Entering Residency, Curriculum Developer’s Guide.2014;
